# Patient-Facing AI System for Symptom Guidance Using Simulated Encounters and Physician Review: Analytical Validation Study

**DOI:** 10.2196/103419

**Published:** 2026-08-19

**Authors:** Anil Patwardhan, Nishant Verma, Poulami Barman, Bingyu Mao, Nan Li, Paul Varghese, Vidya Peri, Lisa Soleymani Lehmann

**Affiliations:** 1 Verily Life Sciences LLC (United States) Dallas, TX United States

**Keywords:** symptom guidance, symptom checker, large language model, triage, analytical validation, digital health, remote care, undertriage, overtriage, simulation

## Abstract

**Background:**

Rapid advances in large language models (LLMs) have expanded interest in patient-facing health applications that support symptom assessment and care-seeking decisions. Although AI-enabled symptom-guidance tools could improve patient navigation and recognition of clinically serious conditions, inappropriate recommendations may result in missed needed care or unnecessary escalation. Structured predeployment evaluation is therefore needed before prospective clinical use.

**Objective:**

This formative predeployment study aimed to analytically validate a locked configuration of the Personal Health Assistant (PHA; Verily Health), an LLM-enabled patient-facing symptom-guidance tool, under controlled conditions using simulated patient encounters and expert physician review.

**Methods:**

We conducted a prospective predeployment analytical validation study using synthetic patient data. The analysis set included 772 synthetic cases generated using published telephone-triage protocols and persona-based, LLM-assisted case generation. Each case included a clinical vignette, structured medical history information, and a simulated multiturn patient conversation. All cases were reviewed by a panel of 5 independent physicians without access to PHA outputs in order to provide level-of-care recommendations. Case-level ground truth was derived from aggregated physician ratings using prespecified plurality and tie-handling rules. Urgent undertriage, nonurgent undertriage, and overtriage were evaluated against prespecified performance criteria. Secondary analyses assessed the clinical appropriateness of PHA-generated possible conditions and escalation, self-care, and laboratory-testing recommendations.

**Results:**

The final analysis set contained 772 cases, including 2 with unevaluable ground truth that were excluded from primary Endpoint denominators. Urgent undertriage was identified in 28 of 406 cases (6.9%, 95% CI 4.6%-9.8%), nonurgent undertriage in 56 of 305 (18.4%, 95% CI 14.2%-23.2%), and overtriage in 40 of 364 (11.0%, 95% CI 8.0%-14.7%). Overtriage met the prespecified criterion of less than 30%, whereas urgent and nonurgent undertriage did not meet their respective criteria of less than 5% and less than 15%, respectively. Secondary findings were generally supportive of the clinical appropriateness of the additional PHA outputs.

**Conclusions:**

PHA met the prespecified overtriage criterion but did not meet the urgent or nonurgent undertriage criteria. The findings identified undertriage as the principal performance gap requiring further product refinement. Because the study used synthetic cases, simulated conversations, and enriched urgency categories under controlled conditions, the results may not reflect performance in real-world populations or deployment settings. Prospective evaluation with actual users in the intended-use population is needed before broader clinical use.

## Introduction

### Background

Rapid advances in large language models (LLMs) have created new opportunities for health care applications that depend on reasoning, synthesis, and decision support. One emerging application is patient-facing symptom guidance, in which digital assistants attempt to guide patients toward an appropriate level of care based on information provided directly by the patient about their symptoms. Symptom checkers and related digital tools have traditionally relied on rules-based systems [1], but newer systems increasingly incorporate AI components [2,3] to support more flexible interaction and richer contextual interpretation. This shift to integrating generative AI may allow patient-facing tools to move beyond branching logic toward more adaptive exchanges that better incorporate symptom context and, when available, relevant medical history.

A reliable AI-enabled symptom-guidance tool could help patients interpret symptoms and make more appropriate care-seeking decisions, improve navigation to the appropriate level of care, reduce unnecessary usage for low-acuity concerns, and enhance recognition of urgent conditions. At the same time, such a tool may also introduce clinically important errors if it provides inconsistent recommendations, fails to recognize cases that warrant urgent or nonurgent follow-up care, or escalates low-acuity cases unnecessarily. A central challenge for these patient-facing guidance tools is minimizing errors related to missed needed care while avoiding unnecessary escalation.

Prior studies of digital symptom assessment show variable and often imperfect triage performance. Evaluations of symptom-assessment apps reveal substantial heterogeneity, with performance commonly reflecting a tradeoff between risk-averse escalation and failure to identify time-sensitive conditions [4-7]. Separate undertriage and overtriage Endpoints distinguish the direction and potential consequences of triage errors; however, these Endpoints have been reported less consistently than overall triage accuracy. More recent evaluations of LLM-based symptom checkers suggest that LLM triage performance may approach that of health care professionals in some settings [2,8]. However, a structured stress test of ChatGPT Health (OpenAI) found that failures were concentrated at the clinical extremes, including undertriage of emergency conditions, while other work has also reported variability in care-seeking advice and limited real-world evaluation [9,10].

The evaluation literature is beginning to move beyond static benchmark cases toward more realistic conversational assessment. For example, SymptomAI used naturalistic symptom conversations from lay users and showed that active symptom interviewing improved differential-diagnosis accuracy compared with user-guided chatbot-style interactions [11]. This finding underscores that patient-facing AI symptom tools should be evaluated as interactive systems, because performance depends not only on model reasoning but also on whether the system elicits clinically relevant information through the conversation. However, differential-diagnosis accuracy and level-of-care accuracy raise different questions. For patient-facing symptom-guidance products, the most immediate safety-critical question is whether the system recommends the appropriate level of care. The Symptom Checker Accuracy Reporting Framework (SCARF) was recently proposed to improve transparency and consistency in studies of symptom-checker accuracy by providing guidance on case selection, reference-standard assignment, evaluation design, and outcome reporting [12]. Although SCARF provides an important reporting foundation, product-specific evaluations of interactive systems must also determine how to represent intended-use conversations, use a sufficiently large ground-truth panel to account for interphysician variability, define clinically meaningful undertriage and overtriage Endpoints, and prespecify performance criteria and sample size requirements.

### Objective

This analytical validation (AV) study was designed to address a methodological gap in the evaluation of patient-facing AI tools. We applied a structured predeployment validation approach to a defined investigational product. The study used prespecified Endpoints anchored on clinically important undertriage and overtriage rates, clinically structured cases rooted in established triage protocols, simulated interactive encounters rather than static vignettes alone, and blinded expert physician review with prespecified ground-truth derivation rules. Because the investigational tool is designed to use historical medical information when available, the synthetic cases also incorporated medical history context.

The primary aim was to determine whether a locked configuration of Personal Health Assistant (PHA) met the prespecified performance criteria for urgent undertriage, nonurgent undertriage, and overtriage. Secondary aims were to assess the clinical appropriateness of PHA’s possible-condition, escalation-guidance, self-care-guidance, and laboratory-testing outputs. This study was intended as an early evidentiary step to determine whether the tool performed acceptably on a large set of known test cases under controlled conditions before proceeding to prospective clinical studies. More broadly, the study illustrates an approach to predeployment evaluation of patient-facing AI systems for symptom guidance that emphasizes clinically relevant error Endpoints, intended-use simulation, and blinded expert review alongside overall performance.

## Methods

### The PHA and Evaluated Configuration

**Figure 1:**
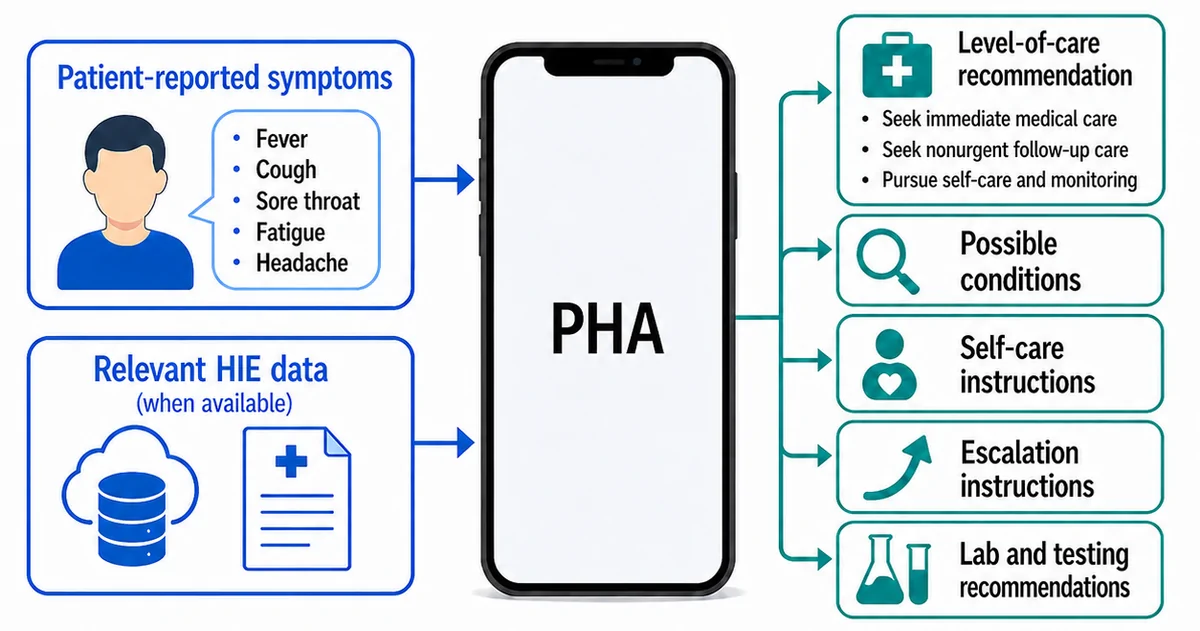
Overview of the Personal Health Assistant (PHA). The PHA is a patient-facing symptom guidance tool that integrates patient-reported symptoms through an interactive conversation and relevant health information exchange (HIE) data. Based on these inputs, PHA generates a level-of-care recommendation (ie, seek immediate medical care, seek nonurgent follow-up care, or pursue self-care and monitoring), and a list of 5 possible conditions. When applicable, it outputs self-care instructions, escalation instructions, and lab/testing recommendations.

The PHA is a patient-facing LLM-enabled symptom-guidance tool that supports conversational assessment of symptoms. It operates as a multiturn agent that combines conversational symptom collection, screening for ineligibility or emergency presentations, retrieval and summarization of relevant medical records from a Health Information Exchange (HIE), iterative refinement through follow-up questioning, and generation of patient-facing outputs. PHA generates a level-of-care recommendation and, for cases assessed as appropriate for nonurgent follow-up care or self-care, also generates possible conditions, escalation guidance, self-care guidance, and recommendations for laboratory testing or other follow-up evaluation. It is intended for adults aged 18 years and older experiencing health-related symptoms in a remote setting and is accessible through a mobile app (Figure 1). Because its outputs may influence how patients interpret their symptoms and whether and when they seek care, careful evaluation is needed before it can be responsibly deployed for general clinical use.

A prespecified version of PHA was locked before execution on the AV test cases, and all results refer to that fixed evaluated configuration. For the version evaluated in this study, PHA was based on the OpenAI GTP-5.4-mini LLM (2026-03-17 model release date). In lieu of the retrieval and summarization of medical records from HIE, this version of PHA required a minimum number of direct patient input fields that included age, sex at birth, social history, immunization summary, known allergies, recent encounter information, chronic conditions, recent laboratory data, and recent diagnoses. These evaluated-version constraints are important for interpretation of the AV results.

### Study Design

**Figure 2:**
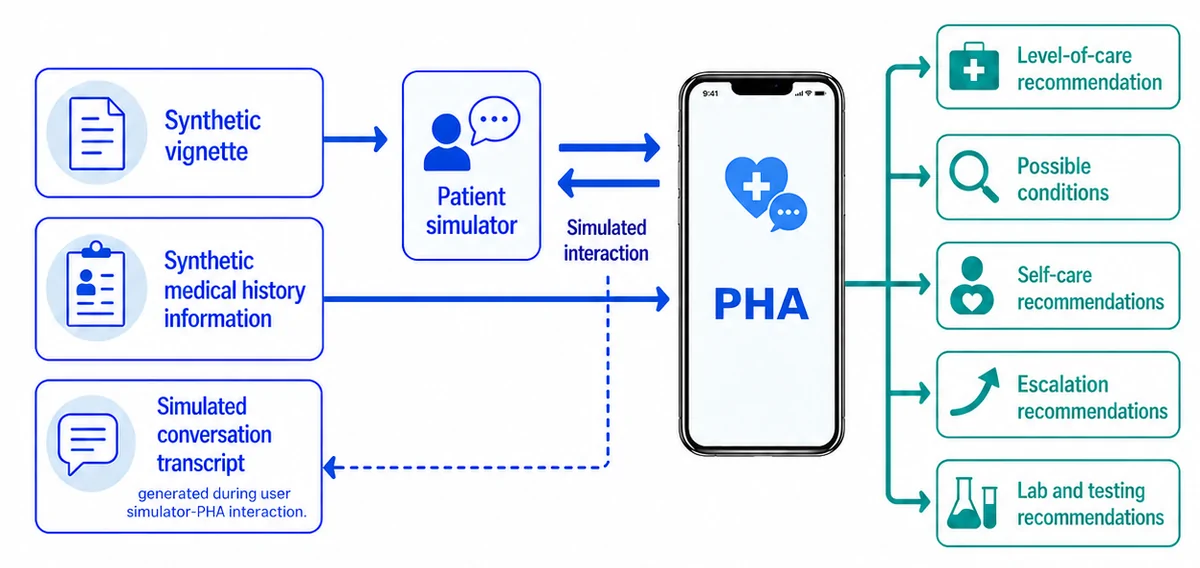
Study workflow for obtaining the Personal Health Assistant (PHA) outputs from synthetic data. A synthetic vignette is used to prompt a patient-simulator, which interacts with the PHA to generate a simulated multi-turn conversation. In combination with medical history information, this conversation enables PHA to generate its outputs.

**Figure 3:**
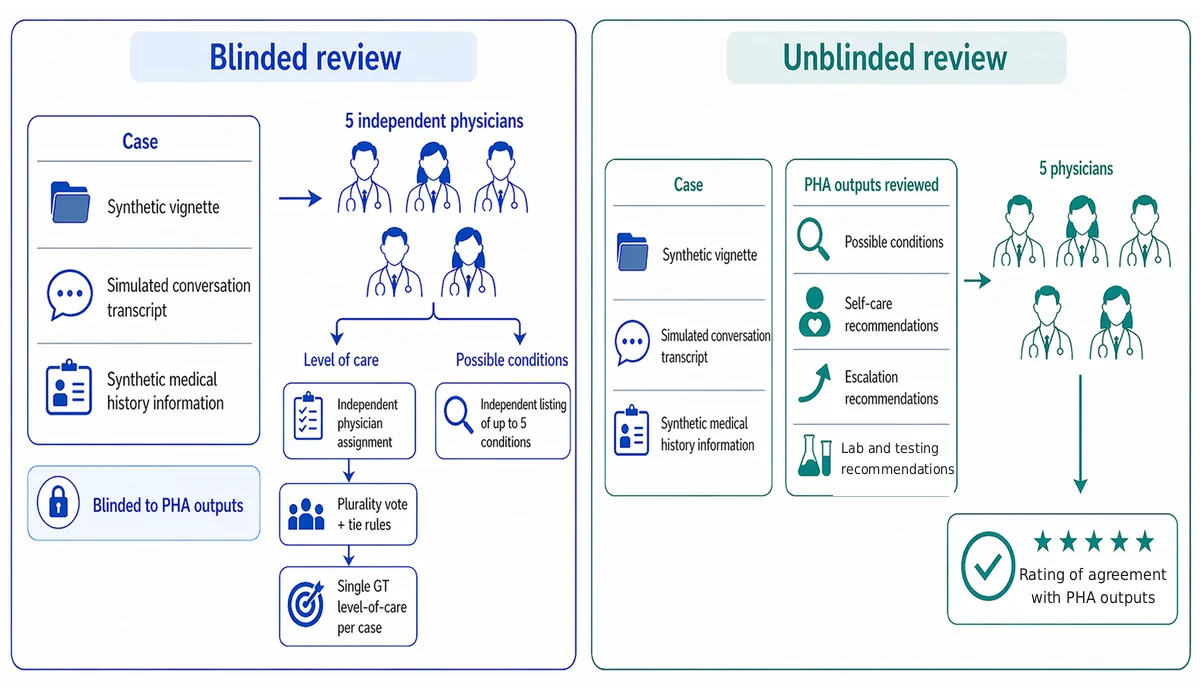
Blinded and unblinded physician review workflow. In the blinded review, 5 physicians independently reviewed only the case materials to provide a level-of-care recommendation and listed up to 5 possible conditions; ground truth for level-of-care was derived by plurality vote with prespecified tie rules. In unblinded review, physicians reviewed the same case materials together with selected PHA outputs and rated agreement with PHA’s outputs of possible conditions and recommendations. GT: ground truth; PHA: Personal Health Assistant.

This study used synthetic patient data to evaluate a locked configuration of PHA under controlled predeployment conditions. Each case combined synthetic patient information, synthetic medical record information, and a simulated patient conversation. PHA outputs and physician-derived reference assessments were collected prospectively for the validation cases. PHA was run on each case to generate patient-facing symptom-guidance outputs (Figure 2), and independent physician reviewers evaluated the same cases using a prespecified grading rubric (Figure 3). The physician review process included blinded and unblinded components to support independent ground-truth derivation for primary Endpoints and appropriateness assessment of selected PHA outputs for secondary Endpoints.

In the blinded review, all cases were reviewed by a panel of 5 independent physicians, resulting in at least 3 physician ratings available per case. Physicians assigned level of care and listed up to 5 possible conditions using the case materials only; ground truth for level of care was derived by plurality vote with prespecified tie rules. In unblinded review, physicians reviewed the same case materials together with selected PHA outputs and rated agreement with PHA’s outputs of possible conditions and recommendations.

### Synthetic Test Case Development and Quality Control

The AV dataset consisted of synthetic test cases representing distinct patient encounters. Each test case contained two related but distinct components: (1) a simulated vignette used to instantiate the patient simulator and (2) a set of structured synthetic medical history fields made directly available to PHA (Multimedia Appendix 1). The vignette summarized the encounter in natural language and included sex at birth, age, social history, family history, medical history, review of systems, history of present illness, current medications, immunizations, allergies, and the chief symptom expressed in patient-facing language. The structured fields made directly available to PHA at the start of the interaction included sex at birth, age, social history, immunization summary, known allergies, chronic conditions, a patient-encounter summary from the prior 12 months, recent laboratory data, and recent diagnoses. PHA then received additional information during its multiturn interaction with the patient simulator.

Briggs’ Telephone Triage Protocols for Nurses, sixth edition [13], was used to assign putative triage urgency to each case and to guide case selection, ensuring adequate numbers of urgent, nonurgent, and self-care cases for evaluation of the primary Endpoints. These protocol-based assignments served only as an initial proxy for urgency during dataset construction; final ground truth (GT) was established subsequently through physician review. A total of 800 synthetic cases were generated in a 400:300:100 allocation of urgent, nonurgent, and self-care categories. Putative triage urgency was not available to PHA or to physician reviewers during evaluation.

Synthetic case generation followed a structured process anchored to the published triage protocols [13] and a persona-based generation approach. Triage protocol logic was decomposed into reusable clinical templates corresponding to different symptom pathways and urgency categories. Patient personas were then varied systematically across social history, family history, medical history, symptom trajectory, and related clinical context to produce a large number of unique but medically coherent scenarios. Structured clinical fields were generated to create plausible longitudinal context for each encounter, and the vignette summarized the broader case narrative in natural language for use by the patient simulator. A detailed description of the case generation process, including example prompts and case structure, is provided in Multimedia Appendix 2.

Cases were created across a broad symptom set selected to support coverage of urgent, nonurgent, and self-care scenarios. Symptom selection was informed by the triage protocols and further enriched with symptoms identified by Verily Health’s clinical team as important to triage correctly, including symptoms that are often benign but may escalate depending on associated history or accompanying symptoms. The full symptom list is provided in Multimedia Appendix 3.

An independent quality control review was performed before evaluation to confirm that the synthetic cases were suitable for testing. A total of 200 cases were randomly selected from the parent set using stratified sampling by putative triage urgency to reflect the parent-set distribution (50% urgent, 37.5% nonurgent, and 12.5% self-care). Review was performed by an independent physician distinct from the physicians later used for GT derivation. The review assessed whether vignettes could reasonably support assignment of a level-of-care category in a remote symptom-guidance setting; whether they were clear and understandable, accurately summarized the key fields, avoided explicit leakage of triage recommendation or other downstream outputs, contained no gross physiologic, anatomic, or medical inconsistencies; and whether the medical history fields were compatible with the vignette.

### Simulated Conversation Component

Each AV case also included a simulated patient conversation. This component was included because PHA was evaluated in an interactive setting rather than on static summary information alone. Simulated conversations were generated using a patient simulator developed previously for conversational evaluation of health care AI agents [14]. In that prior work, the simulator was grounded in real-world electronic health record (EHR)-derived patient vignettes and generated scalable, privacy-preserving, multiturn conversational encounters for development and evaluation of health care AI systems. This prior evaluation assessed more than 500 simulated encounters, with clinician review demonstrating high consistency between simulated conversations and the source vignettes.

For this study, the simulator instantiated each case from the underlying vignette and generated natural-language responses that remained grounded in the source case while disclosing information only as elicited through the interaction. This allowed the simulator to render realistic multiturn encounters rather than simply restating the full case summary at once. A quality control review also assessed whether simulated conversations contained explicit leakage of downstream outputs. When simulated conversation transcripts contained wording that directly implied level of care, that content was manually removed to preserve the independence of physician review.

### Physician Review and Ground-Truth Derivation

Physician reviewers were required to have US medical school training, at least 8 years of posttraining practice experience in the United States, board certification, and consistent practice in emergency medicine or internal medicine, including relevant internal medicine subspecialties. Before formal review began, all reviewers completed study-specific onboarding that included written instructions, orientation to the grading workflow, and a pilot or practice run in which their responses were reviewed for alignment with the grading instructions. Reviewers were instructed to rely on their own clinical judgment based only on the information presented in the study interface and not to use external references or tools during review.

Cases were assigned to reviewers in 2 batches using a partially crossed design. In the first batch, cases were assigned to physicians randomly drawn from a pool of 11 reviewers; in the second batch, reviewers were drawn from a pool of 13. In both batches, assignment was constructed so that each case was assigned to 5 physicians, no case included a duplicate reviewer, workload was distributed approximately evenly across reviewers, and reviewer pairings were balanced so that no specific pair worked together excessively. 332 cases were later reassigned when originally assigned reviewers were unable to complete grading within the required timeframe. Reassignments were distributed across the reviewer pool to preserve workload balance and maintain the partially crossed design. Reviewers worked independently on each case using an online case-review tool [15] and had no access to the responses of other reviewers.

The grading workflow included blinded and unblinded components (Figure 3) that served different evaluation objectives. In the blinded component, all cases were reviewed by a panel of 5 independent physicians, resulting in at least 3 physician ratings available per case. Reviewers assessed the synthetic vignette, simulated patient conversation, and structured clinical fields without access to PHA outputs and independently assigned level of care and up to 5 possible conditions. The blinded level-of-care assessments were used to derive a single ground-truth label per case using plurality vote with prespecified tie rules. In the unblinded component, physicians reviewed the same case materials together with selected PHA outputs and rated their agreement with the PHA possible conditions, escalation guidance, care recommendations, and lab and testing recommendations. This structure allowed independent reference assessment for the primary Endpoint while also supporting evaluation of the clinical relevance of additional patient-facing outputs. Full reviewer instructions and workflow are provided in Multimedia Appendix 4.

GT for level of care was derived from the blinded physician ratings using prespecified aggregation rules. A case required at least 3 evaluable physician ratings to be eligible for GT assignment. Cases with fewer than 3 available ratings were excluded from analysis. For eligible cases, GT was assigned by plurality vote across the urgency categories. When a unique plurality was not present, prespecified tie rules were applied. In general, ties across clinical urgency categories were resolved toward the higher urgency category. For example, in a 2:2:1 split across urgency levels, GT was assigned to the higher of the 2 tied categories. When 2 reviewers assigned a case as unevaluable and 2 assigned the same level-of-care category, the case was assigned as unevaluable.

### Endpoints

PHA generated a case-level level-of-care recommendation for each encounter in 1 of 3 categories: seek immediate medical care, indicating that prompt evaluation in an emergency or otherwise urgent care setting was recommended; seek nonurgent follow-up care, indicating that follow-up with a clinician was recommended but immediate emergency evaluation was not; or pursue self-care and monitoring, indicating that home management and observation were recommended without immediate clinician evaluation. These assignments were compared with physician-derived GT classifications of urgent, nonurgent, or self-care. Primary performance was summarized using 3 population-level mistriage measures derived from that comparison: urgent undertriage, defined as the proportion of urgent GT cases assigned a lower level of care by PHA; nonurgent undertriage, defined as the proportion of nonurgent GT cases assigned to pursue self-care and monitoring by PHA; and overtriage, defined as the proportion of nonurgent or self-care GT cases assigned a higher level of care by PHA.

Secondary Endpoints evaluated PHA outputs beyond the level-of-care recommendation itself. These outputs were intended to provide additional clinical context and guidance, including possible conditions, which summarized conditions that might explain the presentation; escalation guidance, which described when medical attention should be sought if symptoms worsened or failed to improve; self-care guidance, which provided supportive home-management advice; and lab and testing recommendations, which suggested possible follow-up tests or evaluations. Whereas the primary Endpoints assessed whether PHA assigned the appropriate urgency category, the secondary Endpoints assessed whether the supporting explanatory and management content generated by PHA was clinically appropriate for the case. Possible conditions were evaluated by comparison with physician-derived condition lists and by physician agreement assessment. Escalation, self-care, and lab and testing recommendations were evaluated by physician agreement regarding their appropriateness for the case.

### Prespecified Performance Thresholds and Clinical Rationale

Prespecified performance thresholds were defined for urgent undertriage, nonurgent undertriage, and overtriage before analysis. Because no single authoritative consensus defines acceptable mistriage rates for adult, nontrauma, remote symptom-guidance systems, thresholds were selected by triangulating adjacent clinical guidance, published observational evidence, and the practical constraints of remote assessment without physical examination. Thresholds were stratified by urgency level because the clinical consequences of misclassification differ across levels of acuity.

A threshold of <5% was prespecified for urgent undertriage as a conservative, safety-focused benchmark. This threshold was informed by remote-care evidence reviews citing American College of Surgeons Committee on Trauma (ACS-COT) guidance as a proxy reference where more specific adult remote-triage standards are lacking [16], by adjacent studies reporting undertriage rates [17,18], and by the practical recognition that zero urgent undertriage is unrealistic in exam-free remote assessment. A more permissive threshold of <15% was prespecified for nonurgent undertriage, reflecting the lower immediate safety risk of these errors and the wider variability reported in remote and digital triage studies. An overall threshold of <30% was prespecified for overtriage to balance the competing goal of limiting unnecessary escalation while remaining within adjacent benchmark guidance and below many published clinician-led and digital-triage comparators [4,19].

### Statistical Analysis

The unit of analysis was a single synthetic patient-encounter, defined as a case comprising the vignette, simulated conversation, and structured patient information. The analysis dataset included cases with GT assigned from at least 3 physician ratings, including cases assigned an unevaluable GT category. All cases in the analysis dataset received a PHA level-of-care output; cases with unevaluable GT were retained in descriptive summaries but excluded from primary Endpoint denominators.

Primary performance was summarized using 3 population-level mistriage measures: urgent undertriage, nonurgent undertriage, and overtriage. Urgent undertriage was calculated among cases with urgent GT as the proportion assigned a lower level of care by PHA. Nonurgent undertriage was calculated among cases with nonurgent GT as the proportion assigned to pursue self-care and monitoring by PHA. Overtriage was calculated among cases with nonurgent or self-care GT as the proportion assigned a higher level of care by PHA. For each coprimary Endpoint, point estimates and 95% exact Clopper-Pearson CIs were calculated. Performance was evaluated against prespecified thresholds using the upper bound of the 2-sided 95% CI, equivalent to 1-sided exact binomial testing at α=.025.

Supportive analyses examined the robustness of the primary Endpoint results to alternative GT tie rules in which ties were resolved toward a lower level-of-care assignment and to subsets of cases with stronger reviewer agreement, including cases where (1) there were no-ties among physicians; (2) full-consensus subsets, in which all physicians agreed; and (3) cases where all physician reviewers provided an evaluable level-of-care assessment (ie, no unevaluable votes).

A modified analysis population was used for secondary Endpoints related to possible conditions and recommendation outputs. This population excluded cases in which PHA recommended “seek immediate medical care” because these secondary outputs were only generated for cases classified by PHA as appropriate for nonurgent follow-up care or self-care.

For possible conditions, free-text PHA condition terms and physician-derived condition terms were matched using Jaro-Winkler similarity. Four overlap-based metrics were then calculated for each reviewer-case pair: hit rate, indicating whether PHA captured at least 1 physician-listed condition; recall, the proportion of physician-listed conditions captured by PHA; precision, the proportion of PHA-listed conditions captured by physician-listed conditions; and *F*_1_-score, the harmonic mean of recall and precision. Agreement with PHA’s possible conditions output was also summarized as the proportion of cases in which a majority of physician reviewers judged that the PHA list included at least 1 clinically appropriate condition.

For escalation guidance, self-care guidance, and lab and testing recommendations, physician agreement with PHA outputs was rated on a 5-point Likert scale from strongly disagree to strongly agree, reflecting whether the recommendation was appropriate for the case. Physician agreement with recommendation outputs was summarized across individual grader-case ratings using mean Likert scores with 95% bootstrap CIs, together with the distribution of ratings across Likert categories.

The study design targeted approximately 400 urgent GT cases, 300 nonurgent GT cases, and 100 self-care cases, for a total of 800 synthetic cases. Based on simulation using single-sample proportion testing assumptions, this design was expected to provide approximately 71% overall power across the 3 coprimary Endpoints under the planning assumption of 5 physician reviewers per case. Additional details regarding sample size justification, supportive analyses, and secondary Endpoint scoring are provided in Multimedia Appendix 5.

### Ethical Considerations

This study used exclusively synthetic patient cases, synthetic medical history information, and simulated patient conversations. It did not involve living individuals, identifiable private information, or biospecimens. Before study execution, the project was reviewed by the Verily Human Subjects Research Committee, which determined that the activity did not constitute human subjects research requiring institutional review board approval under 45 CFR 46. Because no human research participants or identifiable human data were involved, informed consent was not required.

## Results

### Analysis Sample

A total of 799 cases entered the analytical pipeline from the original 800 synthetically constructed cases, reflecting removal of 1 case because it involved a patient younger than 18 years. During final analysis, 26 cases were identified as duplicates based on identical or near-identical patient summaries representing essentially the same clinical scenario, and 1 additional case was excluded because it involved a patient younger than 18 years. The final analysis dataset therefore contained 772 cases (799 − 26 − 1). There was no loss of cases due to missing PHA outputs, and every case had at least 3 physician ratings available for GT derivation. Of the 772 cases, 770 had an urgent, nonurgent, or self-care GT classification and contributed to at least 1 primary Endpoint denominator; 2 had an unevaluable GT classification and were retained in descriptive summaries but excluded from the primary Endpoint denominators. Mean age was 48.9 (SD 17.6) years, median age was 48 (range 18-85) years, and 410/772 (53.1%) cases were female.

Among the 772 cases, 377 had a PHA level-of-care recommendation other than “seek immediate medical care” and therefore generated secondary outputs for possible conditions, escalation guidance, self-care guidance, and lab and testing recommendations. These 377 cases comprised the modified analysis population used for the secondary Endpoints.

To summarize clinical coverage of the AV dataset, Table 1 groups the cases by presenting-symptom category based on the chief complaint. Categories were assigned from free-text chief symptom descriptions for the purpose of summarizing clinical coverage. For each category, the number of urgent, nonurgent, and self-care cases are shown as determined by physician-derived GT assessments. The full dataset showing symptoms among all cases is available in the Supplementary Materials.

**Table 1:** Presenting symptom categories of Personal Health Assistant cases and physician-derived ground truth.

Presenting symptom category	Representative chief complaints	Urgent, n (%)	Nonurgent, n (%)	Self-care, n (%)	Unevaluable, n (%)	Total, n (%)
Neurologic	Headache, dizziness, weakness, focal neurologic symptoms	142 (18.4)	41 (5.3)	5 (0.6)	0 (0.0)	188 (24.4)
Abdominal, gastrointestinal	Abdominal pain, nausea, vomiting, bowel symptoms	60 (7.8)	40 (5.2)	6 (0.8)	0 (0.0)	106 (13.7)
Musculoskeletal/injury	Back or joint pain, limb pain, swelling, injury	42 (5.4)	35 (4.5)	15 (1.9)	0 (0.0)	92 (11.9)
Cardiopulmonary	Chest pain, dyspnea, cough, wheeze, palpitations	67 (8.7)	20 (2.6)	3 (0.4)	0 (0.0)	90 (11.7)
Genitourinary, reproductive, breast	Dysuria, flank pain, urinary or pelvic symptoms, breast symptoms	28 (3.6)	57 (7.4)	2 (0.3)	1 (0.1)	88 (11.4)
Mental, behavioral	Anxiety, panic-like symptoms, behavioral complaints	8 (1.0)	28 (3.6)	5 (0.6)	0 (0.0)	41 (5.3)
Eye, skin, soft tissue	Eye irritation, rash, pruritus, skin complaints	16 (2.1)	16 (2.1)	8 (1.0)	0 (0.0)	40 (5.2)
Constitutional, general	Fever, chills, fatigue, generalized symptoms	7 (0.9)	15 (1.9)	4 (0.5)	0 (0.0)	26 (3.4)
ENT^a^, oral	Sore throat, ear pain, oral or dental complaints	3 (0.4)	13 (1.7)	3 (0.4)	0 (0.0)	19 (2.5)
Endocrine, metabolic	Thirst, polyuria, heat intolerance, glucose-related symptoms	3 (0.4)	10 (1.3)	3 (0.4)	0 (0.0)	16 (2.1)
Other	Mixed vascular, swallowing, and miscellaneous complaints	30 (3.9)	30 (3.9)	5 (0.6)	1 (0.1)	66 (8.5)
Total	—^b^	406 (52.6)	305 (39.5)	59 (7.6)	2 (0.3)	772 (100.0)

^a^ENT: ear, nose, and throat.

^b^Not applicable.

### Primary Endpoints

The confusion matrix in Table 2 summarizes the distribution of triage assignments by physician-derived GT and by PHA, where rows represent physician-derived ground-truth categories and columns represent PHA level-of-care recommendations. All cases received at least 3 physician ratings; however, 2 cases were excluded from the primary Endpoint denominators because their GT classification was unevaluable. Of the 770 cases with evaluable GT, 406 were classified as urgent, 305 as nonurgent, and 59 as self-care. Across all 772 cases, PHA classified 395 as “seek immediate medical care”, 284 as “seek nonurgent follow-up care”, and 93 as “pursue self-care and monitoring”, with no unevaluable outputs. The 3 coprimary Endpoint results derived from this confusion matrix are summarized in Table 3. Urgent undertriage was identified in 28 of 406 cases (6.9%, 95% CI 4.6%-9.8%), nonurgent undertriage in 56 of 305 (18.4%, 95% CI 14.2%-23.2%), and overtriage in 40 of 364 (11.0%, 95% CI 8.0%-14.7%). A successful Endpoint required the upper bound of the 95% CI to fall below the prespecified clinical threshold. Relative to the prespecified performance thresholds, the overtriage Endpoint met its margin of 30%, whereas both urgent undertriage and nonurgent undertriage exceeded their respective thresholds of 5% and 15%.

**Table 2:** Confusion matrix of physician-derived ground truth versus Personal Health Assistant level-of-care recommendations in the analysis set.

Physician-derived ground truth	PHA^a^: seek immediate medical care, n	PHA: seek nonurgent follow-up care, n	PHA: pursue self-care and monitoring, n
Urgent	378	27	1
Nonurgent	17	232	56
Self-care	0	23	36
Unevaluable	0	2	0

^a^PHA: Personal Health Assistant.

**Table 3:** Primary Endpoint results for Personal Health Assistant level-of-care recommendations in the analysis set.

Primary Endpoint measure	PHA^a^ mistriage events, n	Total cases, n	Rate of mistriage (95% CI)	Prespecified clinical threshold	*P* value
Urgent undertriage^b^	28	406	0.07 (0.5 to 0.10)	0.05	0.752
Nonurgent undertriage^c^	56	305	0.18 (0.14 to 0.23)	0.15	0.941
Overtriage^d^	40	364	0.11 (0.08 to 0.15)	0.30	<.001

^a^PHA: Personal Health Assistant.

^b^Ground truth = urgent; PHA ∈ {seek nonurgent follow-up care; pursue self-care and monitoring}.

^c^Ground truth = nonurgent; PHA = pursue self-care and monitoring.

^d^Ground truth ∈ {nonurgent follow-up, self-care}; PHA more urgent than ground truth.

Most urgent ground-truth cases were correctly recognized by PHA, but a minority were assigned lower-acuity recommendations. Of the 28 urgent undertriaged cases, 27 were assigned to seek nonurgent follow-up care and 1 was assigned to pursue self-care and monitoring. Among the 305 nonurgent ground-truth cases, the dominant error mode was downgrading to self-care: 56 cases (18.4%) were assigned to pursue self-care and monitoring, whereas 17 cases (5.6%) were escalated to seek immediate medical care. Among the 59 self-care ground-truth cases, 23 (39.0%) were assigned to seek nonurgent follow-up care, and no self-care cases were assigned to seek immediate medical care.

### Sensitivity and Subset Analyses

Because GT in this study reflected aggregated physician judgment rather than a single deterministic reference standard, supportive analyses were performed to assess robustness to alternative GT tie handling and to different degrees of reviewer agreement. This design was intentional: plurality-based GT retained clinically ambiguous and borderline presentations in the evaluation, whereas a single-reviewer reference standard would have been less informative and would not have captured interclinician disagreement.

In the primary analysis, ties among level-of-care assignments were resolved toward the higher urgency category, whereas ties involving an unevaluable designation were assigned as unevaluable overall. Table 4 summarizes a sensitivity analysis using the opposing tie rule, in which ties among level-of-care assignments were resolved toward the lower urgency category and ties involving unevaluable ratings were assigned to the level-of-care category with the most votes. This alternative rule produced only small shifts in the Endpoint estimates and did not change the overall conclusion. Under the alternative tie rule, urgent undertriage was identified in 22 of 393 cases (5.6%, 95% CI 3.5%-8.4%), nonurgent undertriage in 56 of 310 (18.1%, 95% CI 13.9%-22.8%), and overtriage in 52 of 377 (13.8%, 95% CI 10.5%-17.7%). As in the primary analysis, the urgent and nonurgent undertriage Endpoints did not meet their prespecified thresholds, whereas the overtriage Endpoint continued to meet its margin.

**Table 4:** Sensitivity analysis of primary Endpoints using alternative ground-truth tie rules.

Primary Endpoint measure	Mistriage events, n	Total cases, n	Rate of mistriage (95% CI)	Prespecified clinical threshold	*P* value
Urgent undertriage	22	393	0.06 (0.04-0.08)	0.05	.75
Nonurgent undertriage	56	310	0.18 (0.14-0.23)	0.15	.94
Overtriage	52	377	0.14 (0.11-0.18)	0.30	<.01

Table 5 summarizes the subset analyses according to reviewer agreement. The plurality-with-no-tie subset included cases in which the GT category had a unique plurality of reviewer votes, with no tie across level-of-care categories. The all-5-evaluable subset included cases in which all 5 reviewers provided evaluable level-of-care assignments (ie, no reviewer assigned the case as “unevaluable”). The full-consensus subset included only cases in which all 5 reviewers agreed on the level-of-care assignment. In the plurality-with-no-tie subset, urgent undertriage was identified in 22 of 393 cases (5.6%), nonurgent undertriage in 56 of 301 (18.6%), and overtriage in 40 of 360 (11.1%). In the all-5-evaluable subset, corresponding rates were 26 of 378 (6.9%), 54 of 279 (19.4%), and 36 of 333 (10.8%). These estimates were closely aligned with the primary analysis. In the full-consensus subset, mistriage rates were lower: urgent undertriage was identified in 3 of 312 cases (1.0%, 95% CI 0.2%-2.8%), nonurgent undertriage in 4 of 92 (4.3%, 95% CI 1.2%-10.8%), and overtriage in 1 of 97 (1.0%, 95% CI 0.0%-5.6%).

**Table 5:** Analysis of under- and overtriage rates in the subset of cases.

Subsets^a^ and primary Endpoint measure			Mistriage events, n		Total cases, n		Rate of mistriage (95% CI)
Plurality (no tie)							
	Urgent undertriage	22		393		0.06 (0.04-0.08)	
	Nonurgent undertriage	56		301		0.19 (0.14-0.24)	
	Overtriage	40		360		0.11 (0.08-0.15)	
All cases with 5 evaluable grades							
	Urgent undertriage	26		378		0.07 (0.05-0.10)	
	Nonurgent undertriage	54		279		0.19 (0.15-0.25)	
	Overtriage	36		333		0.11 (0.08-0.15)	
Consensus (all reviewers agree)							
	Urgent undertriage	3		312		0.01 (0.00-0.03)	
	Nonurgent undertriage	4		92		0.04 (0.01-0.11)	
	Overtriage	1		97		0.01 (0.00-0.06)	

^a^Subset of cases: (1) clear plurality with no tie, (2) all cases with 5 reviewers and no label as “unevaluable,” and (3) cases in which all reviewers agree in the level-of-care assignment (ie, no disagreements).

These analyses indicate that the primary findings were robust to alternative GT tie handling and to reasonable restrictions based on reviewer agreement. More favorable results in the full-consensus subset are consistent with these cases representing clearer, less ambiguous presentations, whereas the primary plurality-based GT approach intentionally retained borderline and clinically variable cases in the evaluation.

### Secondary Endpoints

Secondary Endpoints evaluated the clinical relevance of PHA outputs beyond level-of-care assignment, including possible conditions and recommendations related to escalation, self-care, and labs and testing. These analyses were conducted in the modified analysis population because such outputs were only generated when PHA recommended nonurgent follow-up care or self-care.

For possible conditions, text-based overlap between PHA outputs and grader-provided condition lists showed moderate conceptual alignment. Across the modified analysis population, the mean GT list length was 3.1 conditions per case, and most cases (approximately 70%) had 2 to 4 GT conditions. Across reviewer-case pairs, the hit rate was 0.76, recall was 0.42, precision was 0.26, and *F*_1_-score was 0.31. A high hit rate (76%) indicates that in most cases PHA includes at least one condition aligned with each grader’s thinking. Recall (42%) shows that PHA captures a substantial—but not exhaustive—portion of grader-specified conditions. Precision (26%) is inherently constrained by the fact that PHA always provides five conditions, while GT lists, on average, just over three. PHA commonly proposes additional companion or adjacent conditions beyond what any single reviewer lists. *F*_1_-score (0.31) reflected this balance of partial recall and modest precision.

Among the 377 cases in the modified analysis population, 376 (99.7%) received a positive majority vote indicating that graders agreed the PHA list included at least 1 clinically appropriate condition (95% bootstrap CI 0.992-1.000). This measure reflects physician review of the presented PHA conditions list rather than an independently generated list, and therefore should be interpreted as supportive evidence of clinical appropriateness rather than as an independent accuracy metric.

A similar agreement-based approach was used to evaluate the appropriateness of PHA recommendation outputs beyond possible conditions. Agreement with each recommendation type was rated by physician reviewers on a 5-point Likert scale (strongly disagree, disagree, neutral, agree, and strongly agree). Ratings were grouped as disagree (1-2), neutral (3), and agree (4-5). Table 6 shows the mean Likert score with 95% CI and the number and percentage of ratings in each grouped category. Agreement was highest for escalation and self-care guidance and lower, but still favorable overall, for lab and testing recommendations. Mean Likert scores were 4.41 (95% CI 4.37-4.44) for escalation recommendations, 4.37 (95% CI 4.33-4.41) for self-care recommendations, and 3.99 (95% CI 3.93-4.03) for lab and testing recommendations; corresponding proportions of favorable ratings (Likert 4-5) were 93.9%, 91.4%, and 77.0%, respectively.

**Table 6:** Agreement with Personal Health Assistant escalation recommendations, self-care recommendations, and lab and test recommendations.

Recommendation type	Ratings, n	Likert score, mean (95% CI)	Disagree (1-2), n (%)	Neutral (3), n (%)	Agree (4-5), n (%)
Escalation recommendation	1860	4.41 (4.37-4.44)	61 (3.3)	53 (2.8)	1746 (93.9)
Self-care recommendation	1859	4.37 (4.33-4.41)	79 (4.2)	81 (4.4)	1699 (91.4)
Lab and testing recommendation	1843	3.99 (3.93-4.03)	263 (14.3)	161 (8.7)	1419 (77.0)

## Discussion

### Principal PHA Findings

This study used a structured predeployment analytical validation approach to evaluate PHA, a patient-facing AI system for symptom guidance. The principal finding was that PHA met the prespecified overtriage criterion but did not meet the prespecified criteria for urgent and nonurgent undertriage. The main implication is that unnecessary escalation was relatively limited under the study conditions, but the system still assigned lower-acuity recommendations too often among cases judged to require urgent or nonurgent clinician follow-up. The key performance gap was therefore undertriage rather than overtriage. This is an early evidentiary step for identifying performance gaps under controlled conditions before prospective clinical evaluation. In this case, the results provide product-specific evidence to guide further refinement of the evaluated PHA configuration.

Additionally, when generated, PHA’s recommendation content (escalation recommendations, self-care recommendations, and lab and testing recommendations) was generally viewed by physician reviewers as clinically appropriate, and the possible conditions output showed partial alignment with physician-generated condition lists even though it did not reproduce those lists exactly. These secondary findings should be interpreted as supportive evidence regarding response quality beyond the primary level-of-care recommendations.

### Principal Methodological Contributions

These findings should be understood in the context of staged evaluation for patient-facing clinical guidance tools. Fraser and colleagues [20] argue that such tools should first undergo predeployment laboratory testing and then progress to observational and randomized studies in real-world settings. SCARF further emphasizes transparent reporting of case selection, reference-standard assignment, evaluation design, and outcomes [12]. Consistent with these principles, the present study represents one structured early-stage evaluation of a locked PHA configuration using prespecified clinically relevant error Endpoints, simulated interactive encounters, expert physician review, and explicit ground-truth derivation rules.

Several design choices are central to this contribution. In remote, exam-free settings, the most consequential immediate output is whether the patient is advised to seek care. Accordingly, this study prioritized prespecified urgent undertriage, nonurgent undertriage, and overtriage Endpoints rather than diagnostic accuracy as the primary measure of performance. Our evaluation used clinically structured synthetic cases rooted in established nurse triage protocols, with deliberate coverage of urgent, nonurgent, and self-care scenarios. A key strength was its broad clinical coverage, with cases spanning multiple presenting-symptom categories and diverse patient personas, including scenarios in which acuity depended on medical history, scenarios in which urgency was clarified only through elicited conversational detail, and scenarios in which the presenting symptom was largely unrelated to the available historical context. Finally, GT was based on aggregated blinded judgments rather than a single reviewer, allowing the evaluation to better reflect the inter-reviewer variability that naturally arises in clinically ambiguous and borderline cases. Supportive analyses reinforced this design logic: the overall conclusion did not materially change under alternative GT tie handling, indicating that the primary findings were not driven by a single aggregation assumption. Reviewer-specific mistriage rates also varied, with urgent undertriage ranging from 0% to 16%, nonurgent undertriage from 0% to 41%, and overtriage from 4% to 17% across individual reviewers. The observed spread reinforces that triage assessment is inherently noisy and supports the use of aggregated responses from a panel of physicians as a more robust comparator than 1-2 reviewers.

An additional methodological feature of this study was the use of a multiturn encounter framework rather than static summaries alone, consistent with prior work [11] emphasizing that patient-facing symptom-guidance tools should be evaluated as interactive systems in which clinically relevant information emerges through active elicitation. Symptom severity and other urgency-relevant features may become apparent only through iterative questioning rather than being fully specified at the outset. For a patient-facing symptom-guidance product, the purpose of that interaction is not only to surface plausible conditions, but also to elicit the urgency-relevant information needed to support an appropriate level-of-care recommendation.

### Limitations

While this study provides an early evaluation of an AI-based symptom guidance system, interpretation of primary and secondary Endpoints should account for the limitations inherent in synthetic data.

First, the use of synthetic cases may introduce spectrum and representational bias because such datasets can overrepresent more prototypical presentations and may not fully capture atypical, incomplete, noisy, or culturally and linguistically diverse symptom descriptions encountered in real-world use. Although case generation used structured persona variation and independent physician quality-control review, no formal quantitative demographic or intersectional bias audit was conducted. Notable, however, the dataset was not limited to uniformly clear cases. The more favorable performance observed in the full-consensus subset suggests that cases on which all reviewers agreed represented a clearer subset, whereas the broader dataset included more borderline presentations that generated reviewer disagreement. Second, the use of a single published telephone-triage source also shaped the clinical pathways and urgency assumptions represented in the case set. This source was relevant to the product’s intended use in remote triage and a general nonacute setting, although it may not reflect all clinical practices within such settings.

Similarly, although the patient simulator allows for more naturalistic exchanges than static or prescripted vignettes, it cannot fully capture the variability of real-world encounters. Patient communication is influenced by health literacy and cultural, cognitive, psychosocial, and situational factors, and users may omit information, use unexpected language, or change their description during an interaction. In addition, the simulator was grounded in predefined synthetic source cases; therefore, any errors or structural assumptions in the case data could propagate through the simulated interaction.

A further limitation is that the evaluated PHA configuration was supplied with rich historical medical record information that may exceed what would be available in some real-world use settings (eg, prior patient encounter record recorded in the last 12 months). This creates the possibility of optimistic performance bias due to enriched input information, such that estimated triage performance may be better than would be observed when less contextual information is available. In a real primary care population, for instance, a young patient may not have seen a physician in the last 12 months; however, in this study all user-simulations included this past visit information.

Finally, AV does not establish accuracy against clinical outcomes (eg, patient disposition), real-world safety, effectiveness, or clinical utility. The 400:300:100 urgency allocation was deliberately Endpoint–enriched to provide adequate primary Endpoint denominators and was not intended to represent prevalence in any deployment setting. Urgent and nonurgent undertriage were estimated separately within cases assigned urgent and nonurgent GT, respectively; therefore, these Endpoint–specific conditional rates are not directly determined by the relative prevalence of the 3 urgency categories in the study sample. However, they may still vary if the clinical case mix within an urgency category differs across settings. In addition, because urgency prevalence may differ across direct-to-consumer, primary care, urgent care, emergency, and prehospital populations, aggregate error rates, predictive values, escalation frequency, and operational burden may also differ in practice. Future prospective studies should therefore use a case distribution representative of the intended deployment setting or apply appropriate prevalence-based standardization or reweighting. Such studies should assess actual user interactions, clinical outcomes, utilization, and comparisons with existing triage workflows.

### Conclusion

This study provides a controlled predeployment assessment of a locked patient-facing symptom-guidance AI configuration. PHA met the prespecified overtriage criterion but did not meet the urgent or nonurgent undertriage criteria. The study also illustrates one approach to early-stage evaluation using simulated multiturn encounters, prespecified directional error Endpoints, and blinded physician-panel adjudication. Further product refinement and prospective evaluation in the intended-use population are needed before broader clinical use.

## Data Availability

The datasets generated and analyzed during this study are available from the corresponding author on reasonable request.

## Appendix

Multimedia Appendix 1 Examples of information provided to the patient simulator and information provided directly to Personal Health Assistant (PHA).

Multimedia Appendix 2 Description of case generation process.

Multimedia Appendix 3 Enriched symptoms. The collection of test cases were enriched for the following urgent and nonurgent symptoms identified as being important to triage correctly.

Multimedia Appendix 4 Review instructions and reviewer workflow.

Multimedia Appendix 5 Statistical analysis details.

## Abbreviations

ACS-COT: American College of Surgeons Committee on Trauma
AV: analytical validation
EHR: electronic health record
GT: ground truth
HIE: health information exchange
LLM: large language model
PHA: Personal Health Assistant
SCARF: Symptom Checker Accuracy Reporting Framework
