# Supplementary material for: Patient-Facing AI System for Symptom Guidance Using Simulated Encounters and Physician Review: Analytical Validation Study
Source: JMIR Form Res. 2026 Aug 19;10:e103419. doi: 10.2196/103419 (PMC13539170; doi:10.2196/103419)
Supplement: Supplementary file 1 [file formative_v10i1e103419_app1.docx]

**Multimedia Appendix 1: Examples of information provided to the patient simulator and information provided directly to PHA**

| **Required for Patient Simulator** | |  | **Required for PHA** |
| --- | --- | --- | --- |
| Sex | Female |  | Female |
| Age | 39 |  | 39 |
|  |  |  |  |
| Medical History | Tonsillectomy at age 16. Wisdom teeth extracted at age 22. Ovarian cyst removed at age 30. |  | NOT REQUIRED |
| Family History | Mother has a history of hypothyroidism. Father has a history of high cholesterol. |  | NOT REQUIRED |
| Social History | The patient is a 39-year-old architect who recently started her own firm. She is working long hours and is under considerable stress to secure new clients. She is married and has no children. She and her husband are avid rock climbers, but she hasn't had much time for her hobbies lately. She has been drinking more coffee than usual and often eats takeout meals at her desk. She is a non-smoker and drinks alcohol socially on weekends. She is a very driven and ambitious person, and she is very passionate about her work. She is a very creative and innovative person, and she is always coming up with new ideas. She is a very independent and self-sufficient person, and she is not used to asking for help. |  | The patient is a 39-year-old architect who recently started her own firm. She is working long hours and is under considerable stress to secure new clients. She is married and has no children. She and her husband are avid rock climbers, but she hasn't had much time for her hobbies lately. She has been drinking more coffee than usual and often eats takeout meals at her desk. She is a non-smoker and drinks alcohol socially on weekends. She is a very driven and ambitious person, and she is very passionate about her work. She is a very creative and innovative person, and she is always coming up with new ideas. She is a very independent and self-sufficient person, and she is not |
| Review of Systems | Constitutional: Denies fever, chills, weight loss, or fatigue. HEENT: Denies headache, vision changes, or hearing loss. Cardiovascular: Denies chest pain or palpitations. Respiratory: Denies cough or shortness of breath. Gastrointestinal: Denies abdominal pain, nausea, or vomiting. Musculoskeletal: Denies joint pain or swelling. Neurological: Positive for intermittent tingling in the right hand. Denies weakness, numbness in other areas, or dizziness. Skin: Denies any rashes or lesions. |  | NOT REQUIRED |
| History of Present Illness [i.e. current presenting symptom] | Patient is a 39-year-old female who presents with a two-week history of intermittent tingling in her right hand. The tingling is primarily in the thumb, index, and middle fingers. She reports that the symptoms are worse at night and that she often wakes up with her hand feeling numb. She can relieve the symptoms by shaking her hand. She has been working long hours on her computer and has not been taking as many breaks as usual. She has tried taking over-the-counter ibuprofen without significant relief. |  | NOT REQUIRED |
| Current Medications | None |  | NOT REQUIRED |
| Immunizations | Tdap (2022-09-10), Influenza (2024-10-15), COVID-19 Bivalent Booster (2024-09-20), HPV series completed 2012 |  | Tdap (2022-09-10), Influenza (2024-10-15), COVID-19 Bivalent Booster (2024-09-20), HPV series completed 2012 |
| Allergies | Sulfa drugs (hives), Cats (allergic rhinitis) |  | Sulfa drugs (hives), Cats (allergic rhinitis) |
| Chief Complaint | "My hand keeps falling asleep." |  | NOT REQUIRED |
| Vignette Summary | A 39-year-old female architect presents with a 2-week history of intermittent tingling in her right hand. She has been working long hours on her computer. The tingling is worse at night and is relieved by shaking her hand. She has a history of a tonsillectomy, wisdom teeth extraction, and an ovarian cyst removal. Her mother has a history of hypothyroidism, and her father has a history of high cholesterol. She is allergic to sulfa drugs and cats. She denies any pain, weakness, or other neurological symptoms. A review of systems is otherwise negative. |  | NOT REQUIRED |
| Chronic Conditions | NOT REQUIRED |  | None |
| Last Encounter Notes  within last 12 months | NOT REQUIRED |  | The patient had her annual well-woman exam on January 15, 2025. The visit was routine, and her Pap smear was normal. She and her provider discussed the stress of starting a new business, and her provider encouraged her to make time for self-care, such as exercise and relaxation. Her provider also reminded her of the importance of a healthy diet and regular sleep. No new health concerns were identified, and she was encouraged to continue with her healthy lifestyle and to follow up as needed. |
| Labs within last 3 months | NOT REQUIRED |  | Lipid panel on 2025-07-20 showed borderline high LDL. Comprehensive Metabolic Panel on 2025-07-20 was normal. |
| Previous Diagnoses  within last 12 months | NOT REQUIRED |  | None |
