# Supplementary material for: Patient-Facing AI System for Symptom Guidance Using Simulated Encounters and Physician Review: Analytical Validation Study
Source: JMIR Form Res. 2026 Aug 19;10:e103419. doi: 10.2196/103419 (PMC13539170; doi:10.2196/103419)
Supplement: Supplementary file 2 [file formative_v10i1e103419_app2.docx]

**Multimedia Appendix 2: Description of case generation process**

**Persona-based generation method**

This is a description of the persona-based generation method used to create the synthetic vignettes. This explanation integrates how we vary all the clinical and historical data fields to ensure each case is unique, realistic, and medically sound according to the published triage protocols (Briggs, J. K. (2021). Telephone triage protocols for nurses. Sixth edition. Wolters Kluwer). This creates a large volume of unique medical cases while ensuring each scenario is plausible and aligns with established triage logic and avoids repetition.

**Step 1: Deconstruct Protocols into "Clinical Templates"**

First, a specific protocol from the triage protocol document (e.g., Chest Pain, Headache, Falls) is analyzed and broken down into its essential clinical pathways. Each key symptom cluster or decision point that leads to a specific triage recommendation becomes a reusable "Clinical Template." This ensures that the medical foundation of every vignette is directly tied to the logic in the established protocol.

- Example using the Headache Protocol:
- Template A (Emergency): "Sudden, severe 'thunderclap' headache" +/- stiff neck or neurological changes. -> Triage: Seek immediate medical care.
- Template B (Urgent): Headache with fever and stiff neck. -> Triage: Seek immediate medical care.
- Template C (Non-Urgent): Typical migraine pattern but unresponsive to usual home treatment. -> Triage: Seek non-urgent follow-up care.
- Template D (Self-Care): Mild headache consistent with tension, without red flags. -> Triage: Pursue self-care and monitoring.

**Step 2: Create a Library of Diverse Patient Personas**

Build a large set of distinct patient "personas" by systematically combining and varying background details.

The variables that are mixed and matched for each persona include:

- Social History: This provides the life context.
  - Occupation/Lifestyle: Rotate between sedentary jobs (office worker), physically demanding ones (construction), high-stress professions (lawyer), and different retirement lifestyles (active vs. homebound).
  - Living Situation: Vary whether the person lives alone, with a supportive family, in a care facility, or is homeless. This affects their support system.
  - Health Behaviors: Include details like smoking history, alcohol/substance use, diet, and exercise habits.
- Family History: This adds genetic and social context.
  - Relevant History: A family history of heart disease for a patient with chest pain.
  - Complicating History: A family history of a bleeding disorder for a patient with a head injury.
  - Unrelated History: A family history of psoriasis for a patient with a sprained ankle adds realism.
- Medical History: This details significant past events.
  - Rotate through different past surgeries (appendectomy, cholecystectomy, joint replacement) and events (childbirth, major trauma) to create a believable patient timeline

**Step 3: Diversify Patient Personas**

- In addition to a fixed list of personas, build each one from a set of randomized components. This creates a larger pool of unique patient backgrounds.
  - Social Components:
    - Occupation: Long-haul trucker, gig-economy worker, retired teacher, stay-at-home parent, active-duty military, unemployed.
    - Living Situation: Lives alone, with a large family, in a nursing home, is homeless, recently immigrated.
    - Health Behaviors: Smoker, vegan, marathon runner, recovering alcoholic, sedentary, non-compliant with medications.
  - Historical Components:
    - Chronic Conditions: Combine conditions. A patient might have well-controlled Type 2 Diabetes and GERD, or Atrial Fibrillation and Osteoporosis. Some will have no chronic conditions.
  - Past Medical/Surgical History:
    - Instead of just "no surgeries," use specifics like "appendectomy 20 years ago," "C-section 2 years ago," or "cosmetic surgery last month."
  - Family History:
    - Vary this to be directly relevant (family history of MI in a chest pain case), indirectly relevant (family history of autoimmune disease), or completely unrelated for realism

**Step 4: Integrate Historical Clinical Data to Complete the Persona**

Specific clinical details are added for a documented health journey. These fields are logically linked to the persona's background from Step 2.

- Chronic Conditions Variation: This is a crucial variable that influences risk and presentation.
  - Risk Factors: For a Stroke template, assign a chronic condition like Atrial Fibrillation or Hypertension.
  - Complicating Conditions: For a Shortness of Breath template, the case is very different if the chronic condition is COPD versus Congestive Heart Failure.
  - Unrelated Conditions: A patient presenting with a burn might also have well-controlled hypothyroidism, adding realism.
- Last Encounter Notes Variation: Use this field to establish a baseline.
  - "Stable" Baseline: "Annual physical 6 months ago; no new concerns." This tells us the current problem is acute.
  - "Worsening" Baseline: "PCP visit 1 month ago for high blood pressure; advised medication adjustment." This creates a narrative of an ongoing, unresolved problem.
  - A recent encounter could be for an unrelated issue ("routine skin check"), for the same chronic issue ("hypertension follow-up, BP still high"), or it could be the cause of the new problem ("discharged from hospital 1 week ago after abdominal surgery").
- Labs within last 3 months Variation: Invent labs that are directly relevant to the persona's conditions.
  - Supporting a Condition: A diabetic persona will have a recent A1c (e.g., "7.2%" for controlled, "9.8%" for uncontrolled).
  - Providing a Baseline: For a patient now presenting with fatigue, note, "CBC and TSH 2 months ago were normal," which helps guide the triage process.
  - Absence of Labs: For a young, healthy persona, this field will correctly be "None."
  - Labs will directly reflect the context. A patient on warfarin will have a recent INR. A patient with a new complaint of fatigue might have a note that "CBC and TSH were normal 3 months ago," making the current fatigue more significant. A homeless persona would logically have "None" for recent labs.
- Previous diagnoses within the last 12 months Variation: This shows a patient's recent health journey.
  - Related Illness: A patient with post-herpetic neuralgia will have a Previous Diagnosis of "Herpes Zoster (Shingles) 3 months ago."
  - Recurrent Illness: A patient with a UTI might have a Previous Diagnosis of "Acute Cystitis 8 months ago," establishing a pattern
  - This field will show progression or recurrence. A Previous Diagnosis of "DVT 2 years ago" makes a new presentation of leg swelling much more alarming. An unrelated diagnosis like "Ankle sprain 6 months ago" adds realism.

**Step 5: Synthesize the Vignette and Finalize Clinical Details**

Merge a Clinical Template with the fully-formed Patient Persona. The persona's details bring the generic template to life, and the template provides the acute medical problem.

- History of Present Illness (HPI) Generation: This is the narrative of the template happening to the persona. Vary the story's timeline (sudden vs. gradual), the patient's description of symptoms based on their personality, and what they've already tried for relief.
- Review of Systems (ROS) Generation: The ROS is built to support the most likely diagnosis. It includes positive findings required by the template (e.g., fever and cough for pneumonia) and critical "pertinent negatives" that rule out other serious conditions (e.g., "negative for leg swelling" to make a PE less likely). Include secondary ROS details based on the persona's other chronic conditions to make the picture more complex.
- Apply "HPI and ROS Modifiers" to the Clinical Template . Modify the telling of the medical story to make it unique, even if the underlying problem is similar to another case. HPI (History of Present Illness) Modifiers:
  - Timeline: Was the onset sudden (minutes), subacute (a few days), or chronic (worsening over months)?
  - Patient Behavior: Did the patient ignore the symptom? Did they try a specific home remedy first (e.g., "took Tums for chest pain")? Did a family member push them to call?
  - Symptom Trajectory: Is the symptom constant, intermittent, waxing and waning, or progressively worsening?
- ROS (Review of Systems) Perturbations:
  - To make cases more realistic, introduce secondary "red herring" symptoms or complaints based on the persona's chronic conditions. For example, a patient with a primary complaint of a cough (from a pneumonia template) who also has GERD (from their persona) might have a positive ROS for "heartburn," complicating the clinical picture.

**Step 6: Formatting**

Final form is a google sheet where every row is a patient and fields/columns correspond to the following for either the patient simulator and/or PHA per section 4.3 above:

- Sex
- Age
- Social History
- Family History
- Medical History
- Review of Systems
- History of present illness
- Current medications
- Immunizations
- Allergies
- Chronic Conditions
- Last Encounter Notes within the last 12 months
- Labs within last 3 months
- Previous Diagnoses within the last 12 months
- Vignette summary
- Chief symptom

Additional fields, which are appended as additional columns are used for internal purposes when determining case selection and some information that is useful for subsequent analysis (e.g. standardizing diagnosis to ICD9/10). These columns are available to Biostatistics only:

- likely diagnosis
- ICD9/10 code
- triage urgency recommendation
- rationale for the triage urgency recommendation

**Implementation prompts:**

Environment: Gemini 2.5 Pro, with the following electronic files uploaded:

- Briggs, J. K. (2021). Telephone Triage Protocols for Nurses. Sixth Edition. Wolters Kluwer. These protocols ensure the medical logic is correct.
- Persona.pdf - A single document describing the Persona methodology described above.
- Symptoms.pdf- A single document listing the symptoms that need to be included (Appendix B)

Create a batch of 8 synthetic cases for each of the symptoms in the attachment called ‘symptoms’. To create these synthetic cases, use the persona-based generation method in the attached document (Persona.pdf). These synthetic cases should contain the following information:

Sex

Age

Social History

Family History

Medical History

Current medications

Immunizations

Allergies

Chronic Conditions

Last Encounter Notes within the last 12 months

Labs within last 3 months

Previous Diagnoses within the last 12 months

Chief symptom

Review of Systems

History of present illness

Please vary the social history, medical history, and family histories , review of systems, history of present illness, Chronic Conditions, Last Encounter Notes within the last 12 months, Labs within the last 3 months, and Previous Diagnoses within the last 12 months as described in the Persona method. There should be no duplicated synthetic cases created.

Chart histories should be extensive and diversified. Include exact dates for Labs within the last 3 months and for Last encounter Notes within the last 12 months. Also include exact dates for immunizations.

Last encounter notes in the last 12 months should always have data available and should be extensive. This should be at least a paragraph long assuming that some encounter information is available and should be detailed.

Ensure that the social history and family history are detailed. The social history should be detailed at least one or two paragraphs long.

Ensure that all the cases are for adults 18 years of age or older.

The Chief Symptom should be described as it would come directly from the patient.

I also want a likely diagnosis and triage urgency recommendation. The triage urgency recommendation should fall into the following category: {seek immediate medical care, seek non-urgent follow-up care, pursue self-care and monitoring}. Exclude symptoms where the triage decision would be clearly inappropriate for the category.

Also provide a rationale for the triage urgency recommendation. For the likely diagnosis provide an ICD9/10 code. These likely diagnosis and triage urgency recommendations should align with the triage protocols in the attached document (750714971-Telephone-Triage-Protocols-for-Nurses-2021.pdf). Each synthetic case doesn’t have to use only the symptoms in the triage protocols but should be consistent with how these protocols are used.

I also want a vignette for the patient that summarizes all the information contained in Sex, Age, Social History, Family History, Medical History, Review of Systems, History of Present Illness, Current medications, Immunizations, Allergies, and the chief symptom. This should be at least 2 paragraphs incorporating this information. This summary should be in a field called ‘vignette_summary’. Please verify that the length of this vignette_summary is 2 paragraphs.

I also want the information formatted tab-delimited where each row is a case and the fields map to the individual columns in the sheet. Those columns would be:

Sex

Age

Social History

Family History

Medical History

Current medications

Immunizations

Allergies

Chronic Conditions

Last Encounter Notes within the last 12 months

Labs within last 3 months

Previous Diagnoses within the last 12 months

Likely diagnosis

ICD9/10 code

triage urgency recommendation

rationale for the triage urgency recommendation

Vignette summary

Chief symptom

Review of Systems

History of present illness

The chief symptom, Review of Systems and History of present illness should be the last three columns.

[For ‘seek immediate medical care’ the following additional prompts were used to obtain a larger number of cases]

‘Create more cases with different sets of personas for symptoms that are appropriate but not on the symptoms.pdf list’

‘Create cases for symptoms that are typically considered non-urgent but would escalate to a "seek immediate medical care" recommendation based on standard triage logic. the attached file has some of these symptoms”

Not shown are prompts related to

- Requests for regeneration of cases due to duplication
- Re-request due to a case for the wrong symptom being generated
- Prompts related to formatting issues (e.g. returning csv delimited or pipe delimited instead of tab-delimited, Removing internal line-breaks)
- Sometimes response stops before an entire batch of vignettes is delivered for a specific symptom. So additional prompting used to force continuation
- Request asking for tab-delimited outputs with either a copy icon in the code window or an ‘export to sheets’ tool. Sometimes these spontaneously disappear in the UI interface during some turns
- Request for a case for a specific symptom, when it returned the wrong symptom set
