# Supplementary material for: Patient-Facing AI System for Symptom Guidance Using Simulated Encounters and Physician Review: Analytical Validation Study
Source: JMIR Form Res. 2026 Aug 19;10:e103419. doi: 10.2196/103419 (PMC13539170; doi:10.2196/103419)
Supplement: Supplementary file 3 [file formative_v10i1e103419_app3.docx]

**Multimedia Appendix 3: Enriched Symptoms. The collection of test cases enriched for the following urgent and non-urgent symptoms identified as being important to triage correctly.**

| **Urgent Symptoms** | **Non-Urgent Symptoms** |
| --- | --- |
| Suicidal thoughts or self-harm | Fatigue |
| Hallucinations or psychosis | Excessive sweating |
| Severe anxiety or panic attacks interfering with functioning | Lump in neck |
| Substance abuse or withdrawal symptoms | Lump in breast |
| Sudden or severe headaches | Fever |
| Loss of consciousness | Swelling |
| Sudden vision loss or double vision | Night sweats |
| Severe shortness of breath or gasping | Bruising |
| Sudden severe pain | Swollen glands |
| Severe chest pain or pressure |  |
| Symptoms consistent with heart attack | Cold hands |
| Head injury | Yellow eyes |
| Severe dizziness, confusion, or clumsiness | Jaundice |
| Signs of stroke: paralysis, facial droop, or slurred speech | Heat intolerance |
| Uncontrolled bleeding | Cold intolerance |
| Anaphylaxis | Weight gain |
| Suspected poisoning | Weight loss |
| Severe burns | Difficulty breathing |
| Seizures (especially first-time or prolonged) | Cough |
| Major trauma or significant bleeding | Wheezing |
| Confusion or altered mental status | Shortness of breath |
| High fever in immunocompromised or very young (<3 months)/elderly patients (>65) | Swollen ankles |
| Persistent vomiting | Chest tightness |
| Foreign body ingestion | Palpitations |
| Inability to walk | Blurred vision |
|  | Dry eyes |
|  | Eye pain |
|  | Double vision |
|  | Light sensitivity |
|  | Red eye |
|  | Tearing |
|  | Floaters |
|  | Frequent urination |
|  | Difficulty urinating |
|  | Decreased urine output |
|  | Cloudy urine |
|  | Painful intercourse |
|  | Irregular periods |
|  | Heavy periods |
|  | Missed periods |
|  | Vaginal discharge |
|  | Urinary frequency |
|  | Urinary urgency |
|  | Burning with urination |
|  | Blood in urine |
|  | Incontinence |
|  | Vaginal itching |
|  | Penile discharge |
|  | Scrotal swelling |
|  | Erectile dysfunction |
|  | Painful urination |
|  | Sinus pressure |
|  | Sneezing |
|  | Loss of smell |
|  | Sore throat |
|  | Loss of taste |
|  | Trouble swallowing |
|  | Voice changes |
|  | Snoring |
|  | Hearing loss |
|  | Nasal congestion |
|  | Runny nose |
|  | Hoarseness |
|  | Earache |
|  | Drooling |
|  | Choking |
|  | Sleepiness |
|  | Insomnia |
|  | Frequent waking |
|  | Restless legs |
|  | Dizziness |
|  | Tingling hands |
|  | Tingling feet |
|  | Numbness |
|  | Tremor |
|  | Balance problems |
|  | Clumsiness |
|  | Weakness |
|  | Fainting |
|  | Seizures |
|  | Headache |
|  | Memory loss |
|  | Confusion |
|  | Poor coordination |
|  | Bloating |
|  | Gas |
|  | Belching |
|  | Heartburn |
|  | Constipation |
|  | Indigestion |
|  | Change in bowel habits |
|  | Rectal bleeding |
|  | Abdominal pain |
|  | Black stool |
|  | Loss of appetite |
|  | Vomiting |
|  | Diarrhea |
|  | Nausea |
|  | Clay-colored stool |
|  | Anal itching |
|  | Hiccups |
|  | Anal pain |
|  | Rectal pain |
|  | Neck stiffness |
|  | Back stiffness |
|  | Swollen joints |
|  | Limited range of motion |
|  | Groin pain |
|  | Jaw pain |
|  | Back pain |
|  | Clicking jaw |
|  | Leg cramps |
|  | Leg pain |
|  | Knee pain |
|  | Hip pain |
|  | Wrist pain |
|  | Shoulder pain |
|  | Elbow pain |
|  | Muscle cramps |
|  | Neck pain |
|  | Ankle pain |
|  | Hand pain |
|  | Joint pain |
|  | Foot pain |
