# Supplementary material for: Patient-Facing AI System for Symptom Guidance Using Simulated Encounters and Physician Review: Analytical Validation Study
Source: JMIR Form Res. 2026 Aug 19;10:e103419. doi: 10.2196/103419 (PMC13539170; doi:10.2196/103419)
Supplement: Supplementary file 4 [file formative_v10i1e103419_app4.docx]

**Multimedia Appendix 4: Review Instructions and Reviewer workflow**

***This is a copy of the instructions distributed to all expert-physicians (graders) involved in providing GT for the AV study. This was distributed along with a video describing the grading procedure.***

Your role as a grader in the **PHA Analytical Validation Study** is to help assess the performance of the Personal Health Assistant (PHA), an AI-based tool for patients to understand their symptoms. By providing your own expert medical judgment, your input will be critical in evaluating the accuracy of PHA.

Please leverage your own expert medical judgment in the grading process and do not use external information (e.g. as would come from Google Searches, generative AI, or professional reference materials). The only external information that should be used during the grading process is presented to you through LabelStudio.

Your thoughtful and accurate assessments are vital for this study's success. Thank you for your participation! ✨

**Your Task: A Two-Part Process**

You will complete two distinct phases of assessment for each synthetic patient case. It's crucial that you remain **blinded** to the PHA's outputs during the first phase to ensure your independent judgment is not influenced.

**EVALUATION 1: Blinded Assessment**

In this phase, you will be given:

- a synthetic patient vignette, which is a summary of the patient's information including relevant medical information.
- a transcript of a simulated patient conversation that is meant to mimic the conversation a patient might have with a health care provider (in a remote triage context). In some cases this conversation may be very long. In other cases the conversation may be very brief or the conversation may be one-sided (with substantive input provided only by the patient). All these conversation types are legitimate and you should use the content/substance of the information presented in these conversations as part of your evaluation process.
- Several fields related to historical medical information for the patient including:
  - any known chronic conditions
  - a patient-encounter summary from the last 12 months
  - laboratory results within 3 months prior to the date of the reported symptom
  - any previous diagnosis within the last 12 months

You will **not** see any of the PHA's outputs during this phase.

For each vignette, you must provide a **Triage Urgency Recommendation** by selecting only one (1) of the following pre-specified categories, using a radio-button selection:

**Seek immediate medical care** (this includes urgent and emergency care). Select this if the patient needs medical attention within 24 hours or as soon as possible.

**Seek non-urgent follow-up care**. Select this if the patient should attempt to schedule an appointment with their PCP (not required within 24 hours). This is likely because self-care would be insufficient (e.g., a prescribed drug is needed), or additional examinations and/or diagnostics (e.g., labs) are required to properly assess the patient.

**Pursue self-care and monitoring.** Select this if the patient can safely follow self-care measures and monitor symptoms. If symptoms worsen or do not improve, the patient may need to schedule a clinical appt.

**Unevaluable**. Select this if you find the vignette is not accurate or complete enough to make a triage urgency decision.

IT IS IMPORTANT TO COMPLETE THE SELECTION OF TRIAGE URGENCY RECOMMENDATION FOR EVERY CASE. IF YOU CAN NOT PROVIDE A TRIAGE URGENCY RECOMMENDATION, SELECT ‘**Unevaluable’,** RATHER THEN LEAVING THE CASE UNGRADED.

**For each vignette you must provide a set of Possible Conditions**. Provide a list of up to five possible medical conditions based on the information in the vignette and simulated conversation. (The ordering of possible conditions is not important.)

- This is a free text response and there are 5 free-text response fields to record each of 5 possible conditions.
- You may list up to five possible conditions but are not required to list exactly five.

**It is important that Evaluation 1 be completed before moving to Evaluation 2 (below).** To indicate that you have completed Evaluation 1**, please click the CONFIRM button located at the left/bottom of the window.** This locks your answers before proceeding to the next evaluation. After completing the first phase, you will be unblinded and presented with the PHA's outputs for the same case. This happens as soon as you click the ‘CONFIRM’ button. PHA outputs include five (5) possible conditions and some recommendations. In many cases there may not be any information available in this section (all blank or NA).

For this second Evaluation 2 phase, you will evaluate and provide feedback on the PHA's outputs, **if they are available**. If they are not available (are all NA), then your evaluation for this case is complete and you can proceed to click the ‘submit’ button (bypass Evaluation 2).

**EVALUATION 2: Unblinded Assessment**

Your task is to review a subset of the PHA outputs and assess your level of agreement with them. For this phase, you will evaluate and provide feedback on the PHA's outputs, **if they are available**.

- **Agreement with Possible Conditions**: Review the PHA's list of five possible conditions and answer "Yes" or "No" to the question:

"Based on the provided patient information, are the reported symptoms consistent with at least one of PHA's list of 5 possible conditions?". This requires a response of either ‘Yes’ or ‘No’.

- **Agreement with Escalation Recommendation***: Evaluate the PHA's escalation recommendation. Use the following **5-point Likert scale** to rate your level of agreement:
  - **1 - Strongly Disagree**: The recommendation is inappropriate or potentially harmful.
  - **2 - Disagree**: The recommendation is incorrect or needs major changes.
  - **3 - Neutral**: The recommendation is plausible but there are better options
  - **4 - Agree**: The recommendation is sound and appropriate.
  - **5 - Strongly Agree**: The recommendation is the optimal course of action.
  - Provide a free-text explanation for your rating.

**Escalation Recommendations are clear criteria (such as warning signs or symptoms) outlined for when the patient should seek medical attention either through PCP, urgent care or ER. When PHA's triage decision is self-care, 'when to escalate' is meant for follow-up with PCP. When PHA's triage decision is follow-up with PCP, 'when to escalate' is meant for urgent care / ER.*

**Agreement with Care Recommendation****: Evaluate the PHA's care recommendation. Use the following **5-point Likert scale** to rate your level of agreement:

- **1 - Strongly Disagree**: The recommendation is inappropriate or potentially harmful.
- **2 - Disagree**: The recommendation is incorrect or needs major changes.
- **3 - Neutral**: The recommendation is plausible but there are better options.
- **4 - Agree**: The recommendation is sound and appropriate.
- **5 - Strongly Agree**: The recommendation is the optimal course of action.
- Provide a free-text explanation for your rating.

***Care recommendations are specific steps a patient can take to manage and improve their condition. These are steps for a self-care triage or until the patient sees a physician. Recommendations include lifestyle changes, over-the-counter medications, or other non-invasive interventions. If appropriate, take-home lab tests may be suggested that the patient can perform themselves to monitor their condition.*

- **Agreement with suggested Lab tests**: Evaluate the PHA's proposed follow-up lab tests, if applicable. Use the following **5-point Likert scale** to rate your level of agreement:
  - **1 - Strongly Disagree**: The recommendation is inappropriate or potentially harmful.
  - **2 - Disagree**: The recommendation is incorrect or needs major changes.
  - **3 - Neutral**: The recommendation is plausible but there are better options.
  - **4 - Agree**: The recommendation is sound and appropriate.
  - **5 - Strongly Agree**: The recommendation is the optimal course of action.
  - Provide a free-text explanation for your rating.

**Once you have completed the evaluations described above, please click the ‘Submit’ button to record your responses and proceed to the next case for evaluation.**
