# Supplementary material for: Patient-Facing AI System for Symptom Guidance Using Simulated Encounters and Physician Review: Analytical Validation Study
Source: JMIR Form Res. 2026 Aug 19;10:e103419. doi: 10.2196/103419 (PMC13539170; doi:10.2196/103419)
Supplement: Supplementary file 5 [file formative_v10i1e103419_app5.docx]

## Multimedia Appendix 5. Statistical Analysis Details

### Primary Endpoint Evaluation

Primary performance was summarized using 3 population-level mis-triage measures:

- Urgent undertriage: the proportion of urgent GT cases assigned a lower level of care by PHA,P(PHA = {pursue self care and monitoring, seek nonurgent followup care} | GT = urgent))
- Nonurgent undertriage: the proportion of nonurgent GT cases assigned to ‘pursue self-care and monitoring’ by PHA, P(PHA = pursue self care and monitoring | GT = nonurgent))
- Overtriage: the proportion of nonurgent or self-care GT cases assigned a higher level of care by PHA, P(PHA > GT | GT = {self care, nonurgent})

### Supportive Analyses for Primary Endpoints

Supportive analyses examined the robustness of the primary endpoint results under alternative GT derivation and reviewer-agreement restrictions. These included:

- alternative GT tie rules in which ties were resolved toward a lower level-of-care assignment
- subsets restricted to cases with no tie in reviewer voting; and
- subsets restricted to cases with full reviewer consensus.

These analyses were intended to assess the stability of the primary endpoint estimates to different assumptions about ambiguity in the physician reference standard.

### Secondary Endpoint Scoring: Possible Conditions

Secondary endpoints for Possible Conditions were designed to assess how well PHA captured physician-expected diagnostic concepts. Free-text PHA condition terms and physician-suggested possible conditions were matched using Jaro-Winkler similarity, and 4 metrics were calculated for each reviewer-case pair. These metrics distinguish between the PHA’s ability to recognize clinician-provided conditions and its tendency to propose extraneous conditions, while remaining robust to differences in the number of potential conditions listed by individual reviewers.

For each case, free-text PHA conditions and reviewer-reported possible conditions were mapped to each other using a text matching method (Jaro-Winkler similarity) and 4 metrics:

- Let A denote the set from the reviewers possible-condition list.
- Let B denote the set from the PHA’s most-probable diagnosis.
- Hit Rate = 1{ |A ∩ B| >= 1 } - indicating whether the PHA captured at least one reviewer possible condition
- Recall (R)= |A ∩ B| / |A|— proportion of reviewer terms captured by the PHA
- Precision (P) = |A ∩ B| / |B| — proportion of PHA terms captured by reviewer-terms
- F1 Score: = 2 * P * R / (P + R) (if both > 0); harmonic mean of recall and precision

Metrics were computed per reviewer–case and then summarized at the reviewer level by averaging across all cases. In addition, agreement with PHA’s Possible Conditions output was summarized as the proportion of cases in which a majority of physician reviewers judged that the PHA list included at least 1 clinically appropriate condition.

### Secondary Endpoint Scoring: Recommendation Outputs

For escalation guidance, self-care guidance, and lab/testing recommendations, physician agreement with PHA outputs was rated on a 5-point Likert scale:

- 1 = strongly disagree
- 2 = disagree
- 3 = neutral
- 4 = agree
- 5 = strongly agree

These ratings reflected the reviewer’s judgment that the PHA recommendation was appropriate for the case. Endpoints were summarized using weighted mean Likert scores with 95% bootstrap confidence intervals, together with the distribution of responses across collapsed categories of Disagree (Likert 1-2), Neutral (Likert 3) and Agree (Likert 4-5).

### Sample Size Justification

Sample size planning was based on the 3 co-primary endpoints: urgent undertriage, nonurgent undertriage, and overtriage. Power calculations assumed that each synthetic test case represented an independent patient encounter, such that no patient contributed more than 1 case. Type I error was controlled at 0.05 using a two-sided framework, equivalent to 0.025 for the one-sided margin-based tests used for the primary endpoints. Power was evaluated using single-sample proportion tests comparing assumed PHA performance with the prespecified clinical thresholds.

Endpoints were not assumed to be independent: urgent and nonurgent undertriage were expected to be positively correlated, whereas undertriage and overtriage were expected to be negatively correlated because more conservative triage behavior tends to reduce undertriage while increasing overtriage. To reflect this dependency, simulation assumed a moderate positive correlation of r=0.6 between the 2 undertriage endpoints and a moderate negative correlation of r=-0.5 between undertriage and overtriage. In addition, a variance inflation factor was incorporated to account for reviewer variability and the plurality-vote mechanism used to derive ground truth; this was based on prior feasibility work showing approximately 21% pairwise mismatch among reviewers for level-of-care classification.

The simulation also assumed underlying PHA performance rates of 2.7% urgent undertriage, 10% nonurgent undertriage, and 14% overall overtriage, based on prior feasibility work. Under these assumptions, approximately 400 urgent ground-truth cases would provide 80% power to demonstrate an urgent undertriage rate below the 5% margin, and 300 nonurgent ground-truth cases would provide 86% power to demonstrate a nonurgent undertriage rate below the 15% margin. Because overtriage can occur among both nonurgent and self-care cases, an additional 100 self-care cases were included, yielding 400 total nonurgent or self-care cases for overtriage evaluation; under the assumed 14% overtriage rate, this provided greater than 99% power to demonstrate an overtriage rate below the 30% margin.

Overall, a design target of 800 synthetic cases (approximately 400 urgent, 300 nonurgent, and 100 self-care) was estimated to provide approximately 71% overall power for the 3-endpoint primary objective when using 5 physician reviewers per case.
